# The health equity implementation framework: proposal and preliminary study of hepatitis C virus treatment

**DOI:** 10.1186/s13012-019-0861-y

**Published:** 2019-03-12

**Authors:** Eva N. Woodward, Monica M. Matthieu, Uchenna S. Uchendu, Shari Rogal, JoAnn E. Kirchner

**Affiliations:** 1 Center for Mental Healthcare & Outcomes Research, Central Arkansas Veterans Healthcare System, U.S. Department of Veterans Affairs, 2200 Fort Roots Drive, 152 NLR, North Little Rock, AR 72114 USA; 2 Department of Psychiatry, University of Arkansas for Medical Sciences, Little Rock, AR USA; 3 College for Public Health and Social Justice, School of Social Work, Saint Louis University, St. Louis, MO USA; 4 Health Management Associates, Washington, DC USA; 5 VA Pittsburgh Healthcare System, Center for Health Equity Research and Promotion, Pittsburgh, PA USA; 6 Department of Surgery, University of Pittsburgh, Pittsburgh, PA USA; 7 Division of Gastroenterology, Hepatology, and Nutrition, University of Pittsburgh, Pittsburgh, PA USA; 8 VA Team-Based Behavioral Health QUERI, U.S. Department of Veterans Affairs, North Little Rock, AR USA

**Keywords:** Health disparities, Healthcare disparities, Implementation science, Implementation research, Implementation framework, Health equity

## Abstract

**Background:**

Researchers could benefit from methodological advancements to advance uptake of new treatments while also reducing healthcare disparities. A comprehensive determinants framework for healthcare disparity implementation challenges is essential to accurately understand an implementation problem and select implementation strategies.

**Methods:**

We integrated and modified two conceptual frameworks—one from implementation science and one from healthcare disparities research to develop the Health Equity Implementation Framework. We applied the Health Equity Implementation Framework to a historical healthcare disparity challenge—hepatitis C virus (HCV) and its treatment among Black patients seeking care in the US Department of Veterans Affairs (VA). A specific implementation assessment at the patient level was needed to understand any barriers to increasing uptake of HCV treatment, independent of cost. We conducted a preliminary study to assess how feasible it was for researchers to use the Health Equity Implementation Framework. We applied the framework to design the qualitative interview guide and interpret results. Using quantitative data to screen potential participants, this preliminary study consisted of semi-structured interviews with a purposively selected sample of Black, rural-dwelling, older adult VA patients (*N* = 12), living with HCV, from VA medical clinics in the Southern part of the USA.

**Results:**

The Health Equity Implementation Framework was feasible for implementation researchers. Barriers and facilitators were identified at all levels including the patient, provider (recipients), patient-provider interaction (clinical encounter), characteristics of treatment (innovation), and healthcare system (inner and outer context). Some barriers reflected general implementation issues (e.g., poor care coordination after testing positive for HCV). Other barriers were related to healthcare disparities and likely unique to racial minority patients (e.g., testimonials from Black peers about racial discrimination at VA). We identified several facilitators, including patient enthusiasm to obtain treatment because of its high cure rates, and VA clinics that offset HCV stigma by protecting patient confidentiality.

**Conclusion:**

The Health Equity Implementation Framework showcases one way to modify an implementation framework to better assess health equity determinants as well. Researchers may be able to optimize the scientific yield of research inquiries by identifying and addressing factors that promote or impede implementation of novel treatments in addition to eliminating healthcare disparities.

**Electronic supplementary material:**

The online version of this article (10.1186/s13012-019-0861-y) contains supplementary material, which is available to authorized users.

## Background

Implementation scientists have made much progress in advancing the study and uptake of innovations (e.g., treatments, programs) into clinical care [1–4]. Implementation research has benefitted from comprehensive reviews of implementation theories and conceptual frameworks [5, 6], research designs that are well-suited for implementation research [7–9], more rigorous selection of measures [10–12], and precise terminology for its tools [13, 14]. Yet, the application or utilization of implementation science has not been universally applied across all populations and care systems.

Disparities in healthcare are still a concern in the USA [15–17]. Healthcare disparities are significant differences in access, quality, or outcomes of healthcare between groups not due to selection bias [18, 19]. The group suffering from the disparity is considered vulnerable by proxy of a defining feature (e.g., low income, race, gender) that has led to societal discrimination and stress [17]. As examples of these disparities, a US national report found that poor and low-income households have worse care than high-income households [20]. This report also indicated that overall quality of care varied by geographic region in which a person resided, with some people having lower quality of care based on where they lived [20]. In the US Department of Veterans Affairs (VA), VA patients with mental health conditions and those of lower socioeconomic status had significantly poorer health outcomes than those who did not have mental health conditions or higher socioeconomic status [21]. As a final example of health outcome disparities, in 2013, cardiovascular disease, HIV, and diabetes rates were significantly higher among people of color than white individuals [22]. Implementation researchers could benefit from further methodological advancements to integrate implementation science methods and health disparities methods with the goal to advance health equity for all. Health equity includes fair access to opportunities for optimal health and well-being.

Implementation scientists have started to recognize healthcare disparities as a special case of implementation failure. Researchers have applied implementation science to study healthcare disparities, across topics and settings such as obesity [23], mental illness [24, 25], and primary care services [26]. The US AcademyHealth 9th, 10th, and 11th Annual Conferences on the Science of Dissemination and Implementation in Health highlighted tracks focused on health equity. There is an entire literature on culturally tailored or adapted interventions, and this topic is important in implementation science [27]. The literature on cultural adaptations to interventions is generally restricted to how patient-facing or consumer-facing components of an innovation might change as a result of specific needs for a vulnerable population. There is increasing emphasis on adapting implementation strategies as well. However, there lacks an implementation framework that explicitly addresses health equity determinants to identify and describe some of the adaptations to be made to implementation strategies. Recent work described how health equity researchers might use any implementation framework to identify or understand disparities, or create disparity reducing interventions with targets beyond patient levels. This effort generated a decision tree of effectiveness and/or implementation trial designs for equity researchers to use to expedite the research-to-practice timeline [28]. Overall, the field has learned more about applying existing implementation science evaluation [23] to research with vulnerable populations and also gained knowledge about what implementation processes and strategies may be beneficial for specific groups with certain health conditions. Despite recent methodological advances and emphasis on addressing health equity through implementation science [28], there is no determinants framework that clearly incorporates health equity factors into implementation science. As the next step, a chapter in the most recent edition of Brownson and colleagues’ book on implementation research in health suggested that implementation “models might be modified for application among specific racial/ethnic minorities and other vulnerable populations.” [29]. A comprehensive determinants framework for healthcare disparity implementation challenges is essential to accurately understand an implementation problem and select implementation strategies [5].

A conceptual framework that can explain factors related to uptake of an innovation and disparities in healthcare is critically needed, would propel health equity research forward, and may improve outcomes for vulnerable populations. Although theories that account for individual and environmental factors contributing to health disparities have been applied to health services research [30, 31], these have not included implementation factors. Within implementation research, there is a large repertoire of existing implementation frameworks [6]. Some of these frameworks have the capacity to capture certain health equity determinants. For example, within the Theoretical Domains Framework (version 2) [32], provider knowledge, skills (including competence), social identity, and social influence are just a few of the constructs in which health equity determinants could be embedded. Yet, none of them explicitly focus on or mention health equity. As an example, provider knowledge about a mental health intervention is crucial, and knowledge that mental health is perceived with more stigma within the Latino/a community [33] is equally important in how intervention marketing may be adapted to Latino/a patients. As another example, the construct of power between provider and patient may be assessed because this is an obvious power differential in most healthcare settings. But, the construct of power as exercised in US history of white individuals toward people of color may not be identified through formal implementation assessments in a clinic serving people of color despite the inevitability that racialized power will affect a clinical encounter, how a patient of color perceives other recipients (e.g., clerks), or how the clinic structures, policies, and processes (inner context, local level) operate. There are considerably more examples of how implementation frameworks fall short of assessing health equity determinants. Essentially, because of a lack of explicit focus on health equity in existing implementation frameworks, implementation assessments are unlikely to yield any identification or fruitful information about health equity determinants. Therefore, implementation strategies or interventions cannot be adapted or tailored to address these concerns and thus, it is unlikely health equity is being promoted even if implementation is successful. Indeed, drawing from the field of intervention and public health research, if one applies a set of frameworks, methods, interventions, and measures designed for a general population to a vulnerable population, existing disparities are likely to be maintained or even widened [27, 34]. Therefore, using two widely accepted conceptual frameworks—one from implementation science [18] and one from healthcare disparities [35]—we propose and apply the Health Equity Implementation Framework to assess its feasibility. The Health Equity Implementation Framework presented here is one example of how existing implementation frameworks can be modified for research on vulnerable populations [29].

### Proposal of the health equity implementation framework

#### Implementation science framework: i-PARIHS

The implementation science framework that we modified is a determinants framework [5] and specifies factors relevant to increase uptake of an innovation in healthcare: Integrated-Promoting Action on Research Implementation in Health Services [i-PARIHS; 35]. The i-PARIHS framework explains three levels of implementation elements: (1) *context*, such as system-level mandates that might overwhelm staff or a clinic culture open to changing practices; (2) *recipients*, such as patients who prefer one-to-one visits with their providers or providers with special expertise; and (3) *characteristics of the innovation*, such as negative side effects of a treatment or method of treatment delivery. i-PARIHS also proposes that change must be influenced at each level through a set of implementation strategies known as facilitation, or implementation facilitation [36]. Implementation facilitation is an evidence-based implementation strategy [37] that enables a healthcare context to implement an innovation through relationship building, formative evaluation, problem solving, quality improvement processes, audit and feedback, and many other strategies.

#### Health care disparities framework

Because of the many complicated reasons for healthcare disparities, implementation research efforts should be informed by a framework that explains health disparities at multiple levels, including patients, providers, clinics, and healthcare systems. As such, the Health Care Disparities Framework we integrated with i-PARIHS explains factors underlying differences in healthcare for vulnerable populations [18]. Those factors include (1) *patients*, such as their beliefs about treatment; (2) *providers*, such as time demands on the provider; (3) *the clinical encounter*, which includes the patient-provider interaction and all communication during that visit; and (4) *the healthcare system*, such as a hospital’s commitment to reducing disparities or its culture regarding quality improvement in the delivery of healthcare services.

#### Integrating the two frameworks

There are many reasons we chose to integrate these two conceptual frameworks despite other frameworks in implementation science [6] and healthcare disparities [16, 38, 39]. i-PARIHS has been recently updated with stronger theoretical underpinnings [35] and is user friendly for researchers [3], practitioners, and policy makers. The Health Care Disparities Framework is more specific to the healthcare setting than other disparities frameworks [38]. This framework also allows for identification of disparities in *access* to care in addition to “racially disparate clinical decisions” by healthcare providers (i.e., a *quality* of care problem). Disparate clinical decisions are the focus of other frameworks but have not been well integrated into implementation research [16]. Disparate clinical decisions are significant differences in the actual care provided by healthcare providers based on race or other statuses (e.g., sexual minority, weight) that may be influenced by provider bias against certain groups [40–43].

**Fig. 1:**
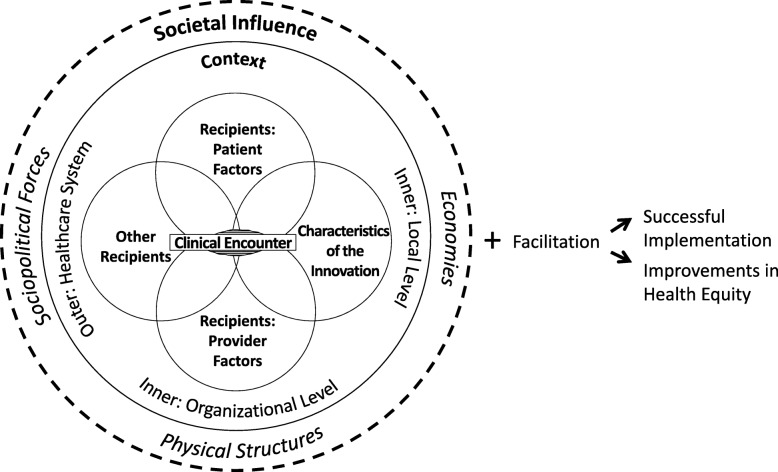
The Health Equity Implementation Framework explains factors relevant to implementation and disparities in healthcare. In this framework, the innovation is delivered in the clinical encounter. We posit that the clinical encounter is an interaction between recipients (e.g., patient and provider) and the innovation itself (e.g., HIV prevention medication), although the interaction could occur in other settings (e.g., between patient and peer navigator). The Health Equity Implementation Framework identified healthcare system factors, broadly, which most closely aligned with the outer context in i-PARIHS. i-PARIHS specified two other levels within context: inner (local—clinic or unit or ward) and inner (organizational—hospital or network). In the Health Equity Implementation Framework, we highlight that societal influence is especially important to consider when assessing all other factors because of the impact society can have on healthcare disparities. Implementation facilitation, or facilitation, is an essential active process to ignite change to any of the elements or factors

Our novel theoretical approach involved integrating both the health care disparities and i-PARIHS implementation science frameworks. We also slightly modified or extended factors related to the clinical encounter (patient-provider interaction), recipients, and societal influence. See Fig. 1 for a depiction of the Health Equity Implementation Framework. When integrated, these frameworks might work synergistically to more fully conceptualize how implementation factors and healthcare disparities factors can both be simultaneously studied and intervened upon.

The Health Equity Implementation Framework is a well-suited theoretical approach to implementation problems that also evince health equity problems because it accounts for factors at multiple levels including those that may be unique to vulnerable populations. Both frameworks are complementary because they attend to elements and factors at multiple levels within the broader environment that are important to successful implementation and address health equity. By attending to multilevel factors in healthcare disparities implementation problems (that is, beyond the patient level), research on health equity can account for the unique factors that vulnerable populations experience because of social and historical marginalization [44]. Similarly, attending to multilevel factors is also essential to promote implementation because the uptake of treatments is greatly affected by multiple variables in complex healthcare systems [45].

In addition, each framework extends the other in certain elements. i-PARIHS provides more specificity to the healthcare system factor identified by the Health Care Disparities Framework to include inner and outer context factors. Inner context factors at the local or organizational level can include leadership support for an innovation, feedback processes, the structure of a system, or any formal policies to embed change within a practice [46]. Outer context factors might include incentives or mandates, and environmental (in)stability of a political, economic, or cultural nature within the healthcare system.

In a complementary fashion, the Health Care Disparities Framework also extends the innovation level of i-PARIHS. i-PARIHS typically defines the innovation level as characteristics related to the treatment itself, such as its usability (e.g., side effects, modes of delivery), its relative advantage over existing treatments, or its trialability for patients [46]. The Health Care Disparities Framework extends the innovation level to also include the clinical encounter, or patient-provider interaction between recipients, which is important to patient satisfaction [47], trust in providers [48], and health outcomes [49]. The clinical encounter might be even more important for patients from vulnerable populations due to preferences unique to these populations [50, 51]. Overall, the integration and modification of both frameworks highlights their unique contributions and expands the scope of each framework as well.

#### Key differences for implementation scientists

The Health Equity Implementation Framework could be used to assess and address health equity and implementation determinants simultaneously. Implementation researchers then have the ability to adapt certain components of the implementation effort to increase the likelihood of improving health equity. Researchers might need to adapt for vulnerable populations either (a) the innovation to be implemented or (b) the implementation strategies used to enhance uptake. To generate a more specific list of determinants to assess than the broad elements in Fig. 1, see the most recent i-PARIHS article or chapter [35, 46] (or whichever implementation determinants framework is preferred) and the Health Care Disparities Framework [18]. 

Using the Health Equity Implementation Framework may also expedite the benefit of research on vulnerable populations compared to traditional implementation frameworks that might (or might not) examine health inequity as a secondary inquiry. In the same way that hybrid effectiveness-implementation designs expedite the translation of research [7], assessing health equity and implementation determinants simultaneously might allow researchers to also shorten the time between when a helpful innovation is ready for dissemination and when it reaches all populations in need of it, equitably.

We do not propose this solely as a new framework that implementation researchers should use, although using this framework for healthcare disparity implementation problems is encouraged, especially if one already uses i-PARIHS. We propose this work to showcase one way to modify an implementation framework to better assess health equity determinants during an implementation effort. Implementation researchers may prefer or need to utilize another implementation determinants framework, such as the Consolidated Framework for Implementation Research or Theoretical Domains Framework; in such cases, health equity determinants can be interwoven into the implementation determinants framework. Below, we describe three key differences for how a modified framework like the Health Equity Implementation Framework extends current implementation determinants frameworks.

##### Attention to the clinical encounter

Although the focus of implementation has predominantly been on context and system factors, health equity challenges require some special attention to the clinical encounter. What occurs in the clinical encounter determines, in part, whether an innovation is delivered, and we argue that this is especially important for healthcare disparities because of unique patient and provider factors in the healthcare of vulnerable groups. One example of the importance of the clinical encounter in implementation is HIV prevention medication (i.e., pre-exposure prophylaxis [PrEP]) for Black and African American men who have sex with men [52–54]. Provider factors such as habits when assessing sexual history interact with patient factors such as mistrust in the medical community [55] in addition to PrEP’s perceived relative advantage given (inaccurate) stigma that it might increase sexual risk behavior [56]. As showcased in this example, even if other elements in the context and healthcare system are facilitators to PrEP implementation, the interaction between recipients and innovation within the clinical encounter (see Fig. 1) may present unique and important implementation barriers for certain healthcare disparity challenges.

##### Societal influence on every determinant

Another key difference for implementation scientists and practitioners to be aware of when handling healthcare disparity challenges is the societal influence downstream on context, recipient, and possibly innovation factors. As depicted in Fig. 1, all factors related to implementation and health equity are affected by societal influence. Societal influence includes the economies, policies, and sociopolitical forces within which patients, providers, and other recipients are living and attempting to be healthy or provide healthcare [57]. We do not propose that societal influence be formally assessed for each implementation project because of the impractical nature of such assessments. Rather, we propose that societal influence be considered when assessing measurable factors and elements as they relate to vulnerable groups involved. An example of societal influence on outer context or the healthcare system broadly might be whether an electronic medical record allows recording patients’ sex at birth and gender identity as one way to identify transgender patients, a group who is significantly marginalized in structural policies and showcases many healthcare disparities [58]. An example of societal influence on the clinical encounter might include the interaction between providers’ unconscious or explicit racial bias and a perception of this bias by a patient of color, given the history of racism in the USA (showcased in the application example in this paper). An example of societal influence on recipient factors might include a lower-income patient who does not adhere to the physical activity regimen due to lack of access to safe walkways, parks, or gyms in their neighborhood. An example of societal influence on characteristics of the innovation might be necessary cultural adaptations (e.g., linguistic alignment with vulnerable populations). The creators of i-PARIHS clarified that “although it may not be possible to directly influence the outer context, it is important to be aware of how the outer context might impact upon local implementation.” [46] So must the implementation scientist treat societal influence outside the healthcare system and its downstream effect on factors related to healthcare disparity implementation challenges that can be assessed and intervened upon.

##### Expanding recipient factors to include determinants specific to health equity

In traditional implementation frameworks, the recipient constructs often focus on knowledge, motivation, or skills of providers or patients related to the healthcare innovation (e.g., total knee replacement). By modifying an implementation framework to include health equity determinants, we can also focus on providers and patients’ knowledge, motivation, and skills that interact with societal influence related to a specific vulnerable group. Recipient factors at the patient level in these implementation health disparity challenges might include beliefs, preferences for treatment, culture and community strengths or limitations, health literacy, and biology [18]. Recipient factors at the provider level might include knowledge about a vulnerable group, attitudes or bias toward that group, and competing demands [18]. Other recipients include individuals who affect the delivery of an innovation, such as a clinic manager, quality assurance administrator, or clerk. An example is provider skill in conducting total knee replacements in addition to provider knowledge and skill discussing this treatment option with Black and African American patients, who do not to receive this helpful innovation at equal rates to white patients [59].

### Application of health equity implementation framework

In this section, we describe an application of this framework to a healthcare disparity implementation challenge—hepatitis C virus (HCV) treatment among Black patients seeking care in the US Department of Veterans Affairs (VA). HCV affects 2.7–3.9 million people in the USA [60] and disproportionately affects veterans [61]. HCV infection can have dire medical consequences, including cirrhosis, liver cancer, and death, and is the leading cause of liver transplants [62]. In the last 5 years, direct acting antiviral (DAA) medications have been approved for patients with all types and stages of HCV [63] These new interferon-free HCV treatment regimens are a considerable improvement on older interferon-based treatments that involved injections, significant side effects, and relatively low cure rates [62]. The newest DAA treatments can cure HCV in greater than 90% of patients [64]. However, DAA treatment is expensive, with a single course of medication costing up to $90,000 [65]. Despite these high costs, the US Department of Veterans Affairs (VA) funds HCV treatment for any beneficiary with HCV who is eligible for VA healthcare services [66]. VA provides a unique setting in which to study HCV treatment uptake. This is because cost and insurance status, common barriers to DAA treatment outside of VA [67], are not limiting factors in this healthcare system.

In VA, there has been an increase in funding and tremendous implementation efforts to increase DAA treatment uptake. VA has significantly increased identification of HCV, linkage to care, and treatment initiation since the introduction of DAAs [68]. By the end of 2016, 59% of Veterans with HCV infection in VA care were treated with DAAs [68]. However, historically, there were treatment disparities in HCV treatment such that Black patients were less likely to receive interferon-based treatment than White patients [69, 70]. In the general US population, the prevalence of HCV is double that of Whites [71]. Effective DAA treatment might have unique implications for Black VA patients given historical disparities [72, 73]; therefore, a specific implementation assessment at the patient level was needed to understand any barriers to increasing uptake of DAA, independent of cost.

Given historical racial disparities for Black patients in VA and outside VA [17, 74], an assessment of implementation factors was needed. The Health Equity Implementation Framework allowed us to account for both traditional barriers to uptake as well as ones that may be specific to Black VA patients. To our knowledge, no data exclusively from Black patients on treatment barriers or facilitators in the DAA era exist. This study was a preliminary implementation assessment consisting of qualitative interviews with Black, Southern, rural-dwelling, older adult male VA patients diagnosed with HCV to examine barriers of and facilitators to starting HCV treatment from the patient perspective. In this study, the focus is on interferon-free DAA treatment, which we refer to henceforth as HCV treatment. Although implementation assessments are typically done with providers and healthcare leadership, an approach to implementation to improve health equity will require engaging patients as key stakeholders as well because of unique needs of vulnerable groups [e.g., 44].

## Methods

### Study design and theoretical approach

The mixed method study design was a partially mixed sequential dominant status design, Quan ➔ QUAL [75]. We used a dominant qualitative methodology to fill gaps in knowledge regarding Black VA patients’ experiences with HCV treatment and to generate hypotheses of explanations regarding the existing healthcare disparity. Our description of the methods follows the Consolidated Criteria for Reporting Qualitative Research (Additional file 1) [76]. Procedures were approved by the Central Arkansas Veterans Healthcare System Institutional Review Board.

### Recruitment and data collection procedures

#### Quantitative data

We used consecutive sampling by reviewing administrative data from VA patient patients’ electronic health records as part of a larger trial of televideo primary care mental health implementation in the rural South [77]. Inclusion criteria at this stage were (a) an outpatient visit at one of six VA community-based outpatient clinics in rural Southern states between October 2015 and June 2016, (b) HCV documented in the electronic health record, and (c) race coded as unknown or any racial category other than white.

#### Qualitative data

Participants were purposively sampled. Prospective participants were sent letters informing them of the study and offering a chance to opt out via telephone or mail within 2 weeks. Then, research assistants randomly recruited prospective participants via telephone calls. Upon receiving verbal consent, VA patients were screened for eligibility, and eligible participants were scheduled for an interview. Inclusion criteria at this stage were (a) self-reported HCV diagnosis and (b) self-reported Black race. The recruitment flowchart is in Additional file 2.

At the scheduled interview time, the research assistant who scheduled the appointment called the participant and introduced the participant to the interviewer via telephone to assist with transferring rapport and then left the call, leaving only the interviewer and participant on the call. The interviewer described study procedures, explained her personal and professional rationale for the study, answered questions, and obtained verbal consent to proceed.

The lead investigator (first author) conducted interviews; she is a white woman, a PhD-level VA clinical psychology fellow at the time, and possessed beginner-level knowledge of implementation science and journeyman-level knowledge of health equity and health psychology. The interviewer’s key assumption was that Black individuals experienced a history of marginalization in US society (e.g., slavery), science (e.g., the Tuskegee syphilis experiment) [78], and healthcare (e.g., provider bias) [40], and that HCV healthcare disparities were partially due to effects of marginalization on Black VA patients and their VA providers.

Interviews were audio recorded. After eight interviews, the interviewer consulted field notes to review for saturation of themes, and engaged in a cycle of interviewing two more participants at a time and consulting field notes until themes were saturated. VA patients were offered VA HCV treatment resource information in their area and a $25 check via mail as compensation for their time.

### Measures

#### Quantitative data

Research assistants collected demographic information via telephone using a quantitative screener to assess eligibility. The screener included questions on race, ethnicity, education, personal annual income, employment, current housing, sexual orientation, gender identity, knowledge of new HCV treatment, and distance traveled to receive HCV care. These questions were queried with a variety of response options documented in Additional file 3.

#### Qualitative interviews

We conducted semi-structured individual interviews with VA patients to identify patient-perceived barriers and facilitators to HCV treatment implementation in VA. The interview guide was based on components of the Health Equity Implementation Framework presented in this manuscript, and allowed the interviewer to follow areas of inquiry as they emerged (see Additional file 4). Iterative drafts of the interview guide were edited by two VA expert qualitative researchers, a VA hepatologist, our VA operational partner, and other VA research team members who identified as either VA patients or Black or rural-dwelling individuals. A final draft was piloted and refined with an HCV-positive individual outside VA and two veterans not diagnosed with HCV. The lead investigator discussed final decisions on interview modifications with research team members over time to enhance specificity of results. The interviewer recorded field notes during and/or after interviews about potential findings and process reflections. All interviews were transcribed verbatim and de-identified and double checked for transcription accuracy. Transcripts were not returned to participants for their feedback (member checking) due to the challenge of re-contacting participants and urgency of expediting treatment for VA patients.

### Analysis

The specific coding strategy we used was directed content analysis because we used our a priori Health Equity Implementation Framework and the interview guide to develop our initial top-level codes [79]. The coding team consisted of three female coders—one PhD psychology fellow with journeyman’s level training in qualitative coding (coder A), one PhD research social worker with expert level training in qualitative coding (coder B), and one master’s level research assistant with novice training in qualitative coding (coder C). The coding team used Atlas.ti software [80].

### Coding process

All transcripts were coded at the top level first (i.e., parent coding). We applied seven top-level codes based on the Health Equity Implementation Framework determinants and reached consensus on six codes with one discrepancy, which was resolved through discussion. All coders coded the first two transcripts to refine the codebook and develop consensus on coding rules. Remaining transcripts were divided between coding pairs for top-level coding (coders A and B coded nine transcripts; coders A and C coded three transcripts). Through this process, we combined two codes and refined our codebook to six top-level codes.

After top-level coding, the data was mined for a specific concept about racial disparities in HCV care (i.e., “disparities”). Disparities were defined as experiences, perceptions, or reasons for differences in treatment between Black and white patients. Coders read through each transcript again scanning specifically for disparities.

Then, coders B and C each coded six transcripts at the second level (i.e., child coding) and coder A reviewed all second level codes. We applied 12 s-level codes. Then, the interviewer initially used the Health Equity Implementation Framework and her field notes to sketch preliminary themes. Within each code, we noted repeating themes mentioned by at least two participants, akin to axial coding in grounded theory [81]. These repeating themes were finalized after data were coded.

## Results

### Participants

We recruited 12 Black, Southern, rural-dwelling, older adult male VA patients diagnosed with HCV. Given that the sample was purposively recruited for racial minority status, over 90% reported being Black or African American. Over half of the sample were retired or disabled with nearly all reporting education beyond high school or equivalent. Over two-thirds of the sample (67%) were aware of the HCV treatment with less than half (42%) having undergone treatment. See Table 1 for full sample characteristics. Interviews lasted between 24 and 66 min (*M* = 46.3, *SD* = 12.7).

**Table 1:** Sample characteristics of 12 Black male VA patients diagnosed with hepatitis C virus (HCV) living in the rural Southern USA

Characteristic	*N* (%)
Age, in years	*M* = 61, *SD* = 8.54, Range = 38–69
Racial background	
Black or African American	11 (92)
Biracial or Multiracial	1 (8)
Current employment status	
Employed full-time	1 (8)
Employed part-time	2 (17)
Disabled	4 (33)
On SSI/SSDI^a^	2 (17)
Retired	3 (25)
Current living situation	
Living on your own	5 (42)
Living with spouse/ domestic partner	3 (25)
Living with friends	1 (8)
Living with parents/family of origin	1 (8)
Living with roommates	0 (0)
Staying with people temporarily	1 (8)
Other (VA homeless program)	1 (8)
Highest education level	
Some high school	1 (8)
High school graduate/GED	6 (50)
Technical school	1 (8)
Some college	4 (33)
Heard about new HCV treatment	8 (67)
Started/completed new HCV treatment	5 (42)
Miles traveled one-way to HCV treatment	*M* = 65.17, *SD* = 53.16, Range = 0–160, *Mdn* = 55

All VA patients denied being Hispanic or Latino, self-identified as cisgender men (not transgender), and self-identified as heterosexual

^a^*SSI* supplemental security income, *SSDI* social security disability insurance

### Main themes

We aligned themes within our Health Equity Implementation Framework elements and present results as facilitators and barriers within each element. Themes with participant quotations are presented by element in Table 2 (facilitators) and Table 3 (barriers).

**Table 2:** Matrix of facilitators: themes and participant excerpts by Health Equity Implementation Framework element for HCV-positive, Black VA patients in the Southern USA

HCV treatment facilitators: matrix of themes		
Health Equity Implementation Framework element	Theme	Excerpt
Innovation factors		
1)	HCV treatment regimen (daily pill for 12 weeks) and cost were acceptable to VA patients	No excerpt: When asked by the interviewer about pill form or cost of medicine at VA, all VA patients denied these being barriers. One exception is that pill form might require some medication management, which is addressed in the barriers section. The VA covered the cost of HCV treatment such that the medications would not cost money for VA patients.
2)	Having the ability to try the treatment first was unimportant because of high cure rate and few side effects.	P 1: *I just want to try [the new HCV treatment]. Like I said, I want to eliminate this, you know. I want whatever works… I want to live a little longer. I have fourteen grandchildren so I want to be around for them.*
Clinical encounter		
3)	Positive clinical encounters regarding HCV occurred when providers explained what HCV was, the new treatment, side effects, next steps, and answered VA patient questions.	P 9: [*The local primary care clinic*] *took some bloodwork and told me* [*I had HCV*]. *they got me in to see the doctor in* [*VA medical center*], *and they went over the stuff with me and they started me on the medication. It happened pretty fast*…*I have no complaints. At first it scared me*…*like being around my grandchildren and stuff*…*and then they explained to me this and that*, *and I can be with the grandkids*. *If I have a cut and they have a cut or something, it can infect my grandchildren*…*And so until I got on that medication make sure I did not visit my grandchildren too often. We talked, and I asked her how long it would take me*—*actually, she called and was setting this stuff up, an appointment up for me when I was there in the office with her*…*I am happy with how it happened*… *They did a lot of bloodwork, and then when they got me in there and got me the medicine and then they gave me the results from last month, they told me about the drop* [*in HCV viral load*] *I had in it last month and I was happy because at least they are taking care of me*. [*The provider*] *talked with me*…*sits down and talks with me every time I get there. I talk to her like I am talking to you. I like her*.
4)	Wait time for an appointment was not an issue for any VA patient.	No excerpt: Once VA patients were offered a follow-up appointment to initiate HCV treatment, each VA patient denied that wait time for that specific appointment was too long. (Concerns about wait time to initiate treatment were expressed and reported in Table 3 findings of barriers).
Recipient factors		
Patient factors		
5)	VA patients hoped there was no racial discrimination in VA	P 1*: I hope color would not make a difference*… *If there’s a cure, then we should all have it. Black or white, it does not matter. Color does not matter*.
6)	VA patients were optimistic about treatment.	P 7: (*When asked about barriers to getting the HCV treatment*): *I do not know because I want to have it. I do not see anything really getting in my way*.
7)	VA patients were eager for more HCV education and outreach.	P 8: *Is* [*the treatment*] *going to work*? *Is it worth all the hassle going over there and getting medicine*? *One thing* [*that would make me likely to get the treatment*] *is if I am guaranteed it*’*s going to work. But I know that there is no guarantee. Another thing I would want to know more information on side effects*… *what I feel would be best would be for the VA to sit down one-on-one to discuss with a doctor that is familiar with the treatment, the side effects*, *and*…*if anybody that do this process get cured*…*you have different sites on the internet but the information is very limited*.
8)	VA patients reported positive trust in some VA providers who encouraged HCV treatment.	P 5: [*There is*] *a* [*VA*] *licensed practitioner*, *who I put all my trust in, she always kept me abreast of everything*... *She offered, she said I seemed to be a good candidate for this treatment*, *if I wanted to accept it. And after she found out about the treatment*, *she informed me*…*she*’*s up to date on everything. I thank God for her*.
		P 2: [*What helped me decide to get treatment was my doctors*] *letting me know and telling me what medicine to take. I will do what the doctors told me to do. I trust them.*
Provider factors		
9)	VA patients perceived that most providers appeared to have a desire to help, reflected by being on time to appointments, explaining information in detail, and acting quickly on follow-up steps	P 5: *I think that they go in and whoever the doctor is talks about the meds*… *hopefully they can get a practitioner like* [*my provider*]. *That makes a big difference when you have somebody that you are seeing that is actually concerned about you. Those that are there to receive a paycheck are not really worried about you, and I do not really find that with her*. *She does not have to be like that. She would call and say this is* [*provider*], *I am calling to check on you and see how you are doing. Many times I might forget to make an appointment*, *but she*’*ll call and remind me to check in with her*. *And that*’*s compassion, you know*. *You do not find that much*.
10)	Some VA providers were perceived as not having or enacting racial biases.	P 5: *I think I am going to give* [*VA providers*] *the benefit on that question* [*about racial discrimination*]. *We have caring physicians, practitioners that are caring enough to know that*…*people of color have been unproportionally less with benefits of medical advancement and they see that this availability now is even more available for minority for the white*, *Caucasian. So now it*’*s been given to them more freely than it was back in say the 70s*.
Context factors		
Inner context: local level (clinics)		
11)	VA clinics offsets HCV stigma in society by protecting patient confidentiality and including HCV-positive patients in other general infectious disease clinics	P 6: (*When asked about HCV stigma getting in the way of treatment*) *No*… *I go to the clinic and the woman calls my name*. *She calls my name, but they do not know what I am going back there for*. *That*’*s why I said I do not have no problems going to the doctor because it*’*s private*.
Inner context: organizational level (VA only)		
12)	VA patients perceived VA used best medicine and genuinely wanted to help VA patients	No excerpt: When asked directly, “do you think the VA uses the best medicine and wants to help Veterans?”, each participant said yes.
Outer context (both VA and outside VA)		
13)	VA patients reported some HCV treatment materials circulating	P 4: *The only information I have*—*have heard it on T*.*V*.*, you know, commercials about Hep C*.
14)	Positive testimonials about HCV treatment made VA patients more likely to want or to try the treatment	P 1: [*My friend*] *just had* [*the treatment*], *and he was telling me that they treated him for twelve weeks with some medicine*…*It was through another clinic*…*his outcome was great*…*They were giving him some kind of pills*, *you know*. *And he told me*, *man*, *matter of fact he told me the other day*, *he said man*, *I got rid of that Hepatitis C in my blood*… *That made me feel great*, *you know*. *That made me want it even more*, *you know*.
15)	Positive testimonials about healthcare in general made VA patients more open to HCV treatment	P 10: *Most of my friends and family go to the hospital and see doctors*…*they go so it*’*s got to be positive*. *I go with my sister twice a week to* [*the doctor*]. *So most of my friends are up in age with me*, *and we all go to doctors and stuff so they go to the hospital*…*I go regularly when I need to be seen*, *you know*.

**Table 3:** Matrix of barriers: themes and participant excerpts by Health Equity Implementation Framework element for HCV-positive, Black VA patients in the Southern USA

HCV treatment barriers: matrix of themes		
Health Equity Implementation Framework domain	Theme	Excerpt
Innovation factor		
1)	VA patients need a medication reminder system to support adherence.	P 6: *I*’*d forget* [*to take the HCV pill*] *because I do not take* [*the pill*] *at the same time*… *I wasn*’*t in the habit of taking it at the same time*…*So sometimes I would not remember if I took it*…*I got one of them boxes*…*You know how they have got the package where you punch them out*. *The packet does not have that*.
Clinical encounter		
2)	Negative clinical encounters occurred when providers did not offer HCV treatment, follow up on results of bloodwork, or explain rationale for decision regarding HCV treatment.	P 1: (*from a VA patient who had not received HCV treatment*, *but tested positive at a recent appointment*) [*My VA provider*] *did tell me about this new drug*…*they had been advertising it on T*.*V*. *She asked if I had seen that commercial on T*.*V*. *about the drug they are using on Hepatitis C*…*I said*, *yes*. *She said do not believe it because it does not work*…*why would they advertise something like this on television if it does not work? Why would a doctor say something like this? It made me feel disgruntled*…*Because I am hearing this and they are saying it cures Hepatitis C in a pill form*, *and then when I talked to her*...*She told me something totally different*...*She could have explained it to me why it does not work*, *you know*.
Recipient factors		
Patient factors		
3)	VA patients lack knowledge of HCV symptoms.	P 4: (*When asked what the VA could do to improve HCV treatment*) *Well*, *I would like to know exactly what is Hep C and what are the symptoms*.
4)	VA patients reported transportation barriers to HCV treatment.	P 10: *I think* [*the VA*] *should pick people up at their house instead of having them meet at a place because like right now I do not have no transportation and the bus leaves at six o*’*clock in the morning so I have to get a ride from somebody around five o*’*clock*…*I do not like bothering people that time of the morning*, *you know*.
5)	VA patients question trust in some providers.	P 5*: I go to the VA doctor*, *and I guess they know best*. *I mean*, *they always want what is best for you*, *right?*
		P 6: [*I would have a problem*] *maybe trusting the VA doctors*. *I guess they really do not treat Veterans like they should and I am just disappointed*. *I do not know*.
Provider factors		
6)	VA patients reported some VA providers lacked expertise about HCV treatment.	P 11: *They need some more specialist*. *My Hepatitis C*, *they should have treated that*. [*The new HCV treatment*] *was free*, *but it just took so long*. *I had to go through all this red tape because it was so confusing*, *not just to me but it was confusing to them too*. *That*’*s why I say they have got to be better trained*, *you know*.
7)	VA patients reported some VA providers appear to have racial biases.	P 7: *Now there was a doctor*…*he mentioned treatment about Hepatitis C*, *but he told me if I smoked marijuana I could not get it*. *I just thought it was old*, *mean man that did not like the fact that I smoke marijuana*…*I wasn*’*t sure that he was going to try to get me treatment for it anyway*…*I take responsibility for* [*smoking*]…*I do not want to make him seem like a bad seller*…*even though I do not think what he was telling me was right*…*I have got mixed feelings about it*… *with all due respect*, *he*’*s an old white man and I am down here in the South and I have reservations about that*. *Not that I do not have trust for the older ones*… *I just got that impression about this old guy*… *because I am in the racial South*, *and if I can remember correctly*, *he did not really touch me*. *He just talked to me*, *and he was kind of grouchy*…*it*’*s like he had an attitude*, *and it could have been some stress from his working*. *I really do not know*, *but an old*, *white man from down here in the South I can easily get the impression that he do not give two cents about black men down here or anywhere*. *Now that might just be my prejudice*, *but I do not think so*.
Context factors		
Inner context: local level (clinics)		
8)	VA patients reported stories about lack of follow-up to VA patient after VA patient tested positive for HCV.	P 1: *What*’*s hard is the doctors in* [*the VA medical center*] *had never started the treatment*, *you know*, *sent it to the* [*community based outpatient clinic*] *here*…*She knew I had this here*. *I did have bloodwork*. *Why has not my treatment appeared that you said you were going to prescribe for me? Why have not they come to the clinic yet*, *and why have not I been told about it?*
Inner context: organizational level (VA only)		
9)	Negative testimonials from family/friends about racial discrimination at VA made VA patients hesitant to go to VA or trust providers.	P 6: [*VA doctors*] *do not concern themselves with things that are important because they treat you just like I have seen in the black community most people will not go to the VA for medical reasons not because the doctors do not know what they are doing because they are not going to get the service they*’*ll get at a private practitioner*. *Well*, *with the blacks there is a lack of trust because most everybody they know – my dad*, *he was a World War II Veteran*, *and they treated him like*, *you know*, *like he was nothing*. *I remember how he was treated*…*I had not gone to the VA clinic for anything*…[*because of*] *I have seen the way they treated him*.
10)	Negative VA experiences are generalized to all VA care.	P 6: [*The VA provider just sits*] *down in there and look at your chart*. *She asks you about your medicines*…*asks about the doses*. *She do not check and see if your condition has changed*…*if you say*, *oh*, *it still hurts*, *she*’*s going to increase the medicine*. *They just write a prescription and you can go*...*I got a cough*. *They do not look down your throat to see what it is causing the problem*…*I can be taking Benadryl for a cough and have throat cancer*. *The services at the VA is piss poor*.
11)	VA patients reported there was not enough HCV treatment at local community-based outpatient clinics and they had to travel to larger VA medical centers.	P 6: *They should not make appointments that you have to go out of town for*…*how can you give me a consult to go to* [*another state*] *when I can barely get to the clinic here? They need to do something with upgrading the infrastructure here*.
Outer context (both VA and outside VA)		
12)	HCV stigma in society made VA patients less likely to reach out to share recovery story or get support in obtaining treatment (e.g., transportation).	P 8: *I did not tell anybody and nobody asked* [*about me getting HCV treatment*]. *I keep to myself*. *I am not an outgoing person*. *People like to talk too much*…*people form opinions about things*. *‘He must have been shooting dope or he must be messing with whatever*.’ *I keep things to myself so that way I will not have that stigma about how I got* [*HCV*].
13)	VA patients reported there are not enough HCV educational materials circulating, especially in rural areas.	P 1: *Now*, [*media*] *should be something they should do something about*. *They should advertise that*. *Let more people know about* [*HCV*]. *Is it a disease? they have done a lot about the Zika virus*. *let us do something about* [*HCV*]… *publications*, *news*, *radio*…*However they can get it across to the people and make them aware of this*.
		P 8: *To be honest with you*, *recently*, *I am just understanding what Hep C really is*…*I always felt Hepatitis C was someone using a utensil or something that wasn*’*t clean or a blood transfusion*. *Previously*, *that*’*s where I felt Hepatitis came from*…*I wasn*’*t really aware*…*I grew up in a rural area*. *Drugs were not relevant there*.

We identified 15 facilitators of HCV treatment uptake for Black, male, rural, Southern-dwelling VA patients. Innovation facilitators were (1) that the HCV treatment regimen (daily pill for 12 weeks) and cost were acceptable to VA patients and (2) the ability to trial the treatment first was unimportant because of high cure rate and few side effects. Clinical encounter facilitators included a description of (3) positive encounters (see Table 2 for detail) and that (4) VA patients denied any concerns about wait time for an appointment once they were offered HCV treatment. At the recipient level, patient facilitators were that (5) VA patients hoped there was no racial discrimination in VA, (6) were optimistic about treatment, (7) were eager for more HCV education and outreach, and (8) reported positive trust in some VA providers. Provider facilitators were that (9) VA patients perceived that most VA providers appeared to have a desire to help and (10) some VA providers were perceived as not having or enacting racial biases. Facilitators in the inner context were that (11) VA clinics offsets HCV stigma by protecting patient confidentiality and (12) VA patients perceived VA used best medicine and genuinely wanted to help VA patients. Facilitators in the outer context were that (13) VA patients reported some HCV treatment materials circulating, (14) positive testimonials about HCV treatment made VA patients more likely to want the treatment, and (15) positive testimonials about general healthcare made VA patients more open to HCV treatment.

We identified 13 barriers to HCV treatment. The innovation barrier was that (1) VA patients needed a medication reminder system to support adherence. The clinical encounter barrier was (2) negative clinical encounters, characterized by providers not offering the new HCV treatment, lack of follow up on results of bloodwork to detect HCV, or no rationale for decisions regarding variations in HCV treatment. Recipient barriers at the patient level included (3) VA patients lacked knowledge of HCV symptoms, (4) VA patients reported transportation barriers to HCV treatment, and (5) VA patients questioned trust in some VA providers. Recipient barriers at the provider level were that (6) VA patients reported some VA providers lacked expertise about HCV treatment and (7) VA patients reported some VA providers appear to have racial biases. Inner context barriers included (8) stories about lack of follow-up to VA patient after testing positive for HCV, (9) that negative testimonials from family /friends about racial discrimination at VA made VA patients hesitant to go to VA or trust providers, (10) negative VA experiences were generalized to all VA care, and (11) not enough HCV treatment at local community-based outpatient clinics such that VA patients had to travel to larger VA medical centers. Barriers in the outer context were that (12) HCV stigma in society made VA patients less likely to reach out to share their recovery story or get support in obtaining treatment and that (13) VA patients reported that there are not enough HCV educational materials circulating, especially in rural areas.

## Discussion

The goal of this paper was to propose an implementation science framework (i-PARIHS) [46] integrated with a health disparities framework (Health Care Disparities Framework) [18]. The resulting Health Equity Implementation Framework consisted of relevant determinants to assess and address related to implementation problems that might contribute to healthcare inequities between vulnerable and reference groups. We applied the Health Equity Implementation Framework to a preliminary assessment of implementation barriers to and facilitators of HCV treatment among Black VA patients—a group for whom HCV is diagnosed at significantly higher rates compared to white VA patients [82]. We used this specific example because it represented a unique opportunity given that a newer, safer, more effective HCV treatment was made available in VA before this study. This framework could be adapted for other vulnerable individuals who face health disparities (e.g., people without or transitioning homes; sexual minority individuals; people with visual disabilities). One challenge in applying this to other vulnerable groups is the knowledge of what to assess in each domain unique to that group. Thus, the role of engaged stakeholders who work regularly with those vulnerable groups and the role of patients and other healthcare consumers (e.g., family) with lived experience is essential in designing, executing, and interpreting results from an implementation assessment. In fact, the use of community engagement is emerging as one approach to ensure implementation researchers assess determinants unique to certain vulnerable groups [83, 84].

### Advantages

Applying the Health Equity Implementation Framework allowed us to assess barriers and facilitators specific to this vulnerable group while simultaneously capturing barriers that were either independent of, or co-occurring with, typical implementation barriers. Overall findings indicated that there were typical implementation barriers that likely would have been reported by any patient group. One example of a typical implementation barrier at the innovation level included wanting blister packaging for HCV antiviral medication so that patients could more easily keep track of daily medication adherence. Another example of a typical implementation barrier at the organizational level was that difficulties in other VA services made VA patients less likely to access any treatment through VA.

We also identified implementation barriers likely unique to Black VA patients. One example of a unique implementation barrier at the provider level was that some VA patients perceived that some providers might have racial biases that affected their patient-provider interactions. Another example of a unique implementation barrier at the organizational level was that testimonials from others about perceived or enacted racial discrimination at VA made Black VA patients less inclined to access any treatment at VA. By understanding both implementation barriers typical to any patient group and those likely unique to Black patients, we understand the additional burden for Black VA patients living with HCV. Our findings might partially explain why healthcare disparities exist between Black and white individuals—typical implementation barriers are likely applicable to both groups (e.g., difficulty accessing other services) while the unique implementation barriers are likely applicable only to Black VA patients (e.g., testimonials about racial discrimination). Healthcare disparities are certainly concerns in systems outside VA [16, 85]; therefore, the Health Equity Implementation Framework would likely be helpful in any healthcare setting.

If we had not used the Health Equity Implementation Framework, we would have designed the interview guides without systematic attention to possible sources of healthcare disparities. We also would have collected different data from Black VA patients, and ultimately, analyzed and interpreted the scope of barriers and facilitators without much attention to how unique factors relevant to Black VA patients affect the implementation challenge of widespread HCV treatment. For an implementation challenge with a vulnerable population, using the Health Equity Implementation Framework would allow implementation scientists to better explain varying levels of uptake between groups [86]. The framework would also allow adapting implementation strategies to barriers that might be unique to the vulnerable group [87], thereby increasing uptake overall and improving health equity. Health equity researchers looking to incorporate more implementation science into their work may also benefit from the Health Equity Implementation Framework or our other work using a study design decision tree [28].

Another advantage of using the Health Equity Implementation Framework was that we collected data on facilitators of implementation. These can be helpful in several ways. Facilitators can be cited as positive reinforcement at a local site when giving feedback on HCV treatment implementation; highlighting facilitators as well as barriers is recommended during audit and feedback. It can boost provider performance to highlight facilitators (or positive feedback) for tasks that promote quality improvement changes in treatment [88]. Knowledge of facilitators also confirms processes, policies, or cultures to maintain—areas that do not require intervention—which leads to more efficient use of implementation and quality improvement resources. Implementation strategies can also be tailored by using facilitators—some of which have already been utilized for this implementation challenge in VA [68]. An example from this study is the patient-level facilitator that VA patients were eager for more HCV information. Given that the desire for treatment information is present and information is a necessary component of behavior change (i.e., choosing to initiate HCV treatment) [89], implementation strategies were used to engage consumers such as through TV ads or mailed letters on new HCV treatment or educational meetings about HCV treatment for HCV-positive patients [13].

### Challenges

There were some challenges in our application of the Health Equity Implementation Framework during the study. First, by designing the interview guide with this framework in mind, we were systematically prompted to assess not only known sources of barriers/facilitators from other studies on HCV treatment but also known sources of barriers/facilitators from other studies on receipt of healthcare by Black patients. In other words, we had to invest additional effort to ensure we were asking participants about potential implementation factors relevant to two topic areas (HCV treatment and healthcare for Black patients) rather than one. This is an investment we believe is necessary to fully understand implementation problems that also cause healthcare disparities. Nevertheless, it is an important investment to consider when planning study timelines, participant burden, and staffing to ensure adequate expert knowledge is available.

A second challenge was that when asking directly about potential factors related to healthcare disparities, such as differential healthcare treatment by race, most participants denied these factors. Yet, during the interview, participants reported several findings about healthcare disparity implementation factors. Participants would qualify statements as being unique to their racial group (e.g., “In the African American community…”) or would discuss racial discrimination in the context of another general question about barriers. We have provided our interview guide in Additional file 4 to showcase questions specifically about disparities.

A final challenge of this work is that we did not evaluate if there was a reduction in healthcare disparity in our application of the framework. It would be helpful if future research compared the Health Equity Implementation Framework to another implementation framework on the implementation outcomes between vulnerable and reference groups. In addition, a validation study of this framework is needed.

### Limitations

We cannot ascertain whether participants would have shared information specific to this healthcare disparity and Black VA patients even if we had not asked directly about them. It is possible that although asking directly about disparity factors did not elicit any information immediately, it primed participants to think about this topic and feel safe sharing information related to disparities. Future researchers might consider a methodological study to answer this question in the future by comparing results using two different interview guides on matched samples representing a vulnerable group—one interview during which healthcare disparity implementation factors were assessed directly and one interview during which there was no specific assessment of healthcare disparity implementation factors. Also, our results about perceived racial discrimination may be unique to the geographic setting from which these participants were interviewed.

Another limit of the preliminary application of this framework is that we did not assess the degree to which implementation facilitation would be adapted to this healthcare disparity challenge. Implementation facilitation is an essential process to create implementation change, according to i-PARIHS [46]. However, the application in this manuscript focused on the determinant elements of the i-PARIHS framework. Implementation facilitation may uncover special barriers and facilitators in healthcare disparities challenges, but more work is needed on how facilitation or any implementation strategy might be adapted for healthcare disparity implementation challenges.

## Conclusions

This manuscript provides a starting point for future work in better conceptualizing the application of implementation science to address healthcare disparities. The Health Equity Implementation Framework demonstrated feasibility to design survey materials and interpret results. The Health Equity Implementation Framework could be used to underlie the recruitment targets and methods, sampling, study design and data collection tools, type of analysis, and interpretation of results in health services and implementation research. For implementation research, proper planning is required to ensure that participant burden is minimized given assessment of healthcare disparities in addition to typical implementation factors, and that expert knowledge and research team skills are adequate for vulnerable populations. By using the Health Equity Implementation Framework, implementation scientists can optimize the scientific yield of their research inquiries by capturing and addressing information related to both implementation at large and healthcare disparities, should they exist in selected health service research areas. We hope that scholars will apply and refine the framework we proposed.

## Additional files

Additional file 1: COREQ (COnsolidated criteria for REporting Qualitative research) Checklist. This checklist outlines how this manuscript followed standard reporting of our qualitative research. (PDF 489 kb)

Additional file 2: Recruitment Flowchart. This flowchart depicts the number of individuals contacted for participation in the research, exclusions, opt outs, attrition, and the final sample. (DOCX 56 kb)

Additional file 3: Quantitative Screening Questions. These questions were used to screen participants via telephone for eligibility in the preliminary study. (DOCX 15 kb)

Additional file 4: Interview Guide for HCV Treatment Implementation Assessment Informed by Health Equity Implementation Framework. This is the interview guide used in the preliminary study with patient participants that was aligned with the Health Equity Implementation Framework and showcased questions specifically about disparities. See the discussion section for limitations and advantages of using those questions about disparities. (DOCX 26 kb)
