# Supplementary material for: The health equity implementation framework: proposal and preliminary study of hepatitis C virus treatment
Source: Implement Sci. 2019 Mar 12;14:26. doi: 10.1186/s13012-019-0861-y (PMC6417278; doi:10.1186/s13012-019-0861-y)
Supplement: Supplementary file 2 — Recruitment Flowchart. This flowchart depicts the number of individuals contacted for participation in the research, exclusions, opt outs, attrition, and the final sample. (DOCX 56 kb) [file 13012_2019_861_MOESM2_ESM.docx]

**Additional file 2**

Recruitment Flowchart

*
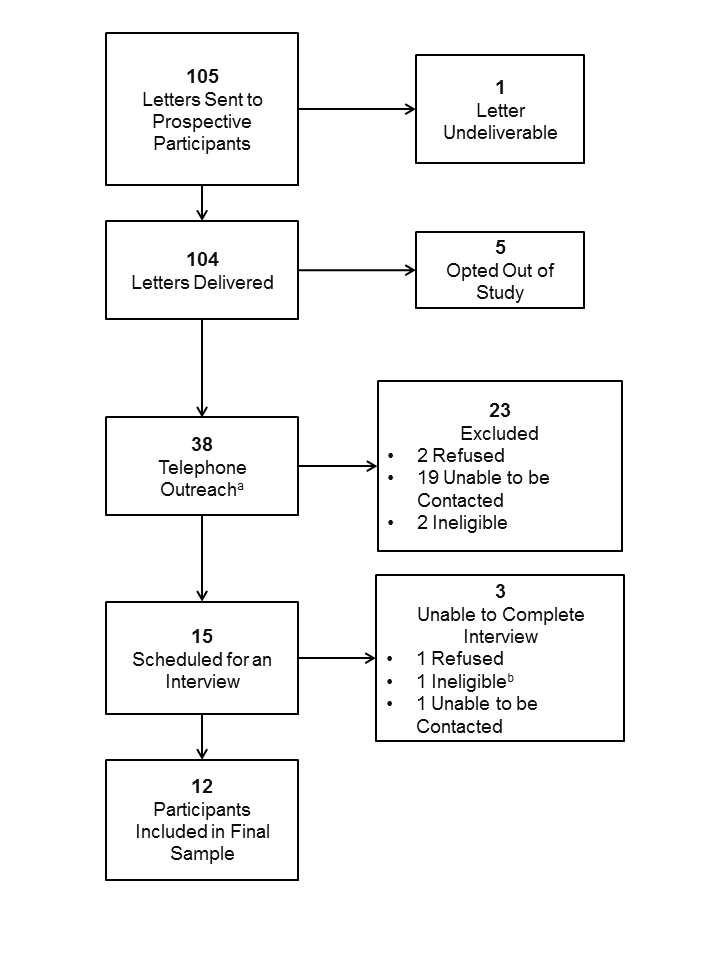
*

Note*. a*. Number of participants contacted by telephone was based on saturation of themes; once saturation was reached, telephone recruitment stopped. *b.* Despite endorsing being diagnosed with Hepatitis C during telephone outreach screening, this VA patient denied he was diagnosed with Hepatitis C at the time of the interview and instead, reported he was diagnosed with Hepatitis B.
