# Supplementary material for: The health equity implementation framework: proposal and preliminary study of hepatitis C virus treatment
Source: Implement Sci. 2019 Mar 12;14:26. doi: 10.1186/s13012-019-0861-y (PMC6417278; doi:10.1186/s13012-019-0861-y)
Supplement: Supplementary file 3 — Quantitative Screening Questions. These questions were used to screen participants via telephone for eligibility in the preliminary study. (DOCX 15 kb) [file 13012_2019_861_MOESM3_ESM.docx]

**Additional File 3.**

Quantitative Screening Questions

I would like to start by asking a few general background questions about you.

**1. What is your age**: ___________ years?

**2. “Which of these best describes your racial background? I’ll read options and you can say any that apply.”**

**(check all that apply).**

- 1 – American Indian or Alaska Native
- 2 – Asian (includes East Indian); please specify _______________
- 3 – Native Hawaiian or other Pacific Islander
- 4 – Black or African American
- 5 – White/Caucasian
- 6 – Bi or Multi-racial, please specify:_________________
- 7 – Other

**3. What is your current employment status? I’ll read options and you say what fits best.**

- 1 – Employed full time
- 2 – Employed part-time
- 3 – Unemployed
- 4 – On sick leave
- 5 – Disabled (if yes, ask is this a VA Service-connected disability? (yes/no)
- 6 – On SSI/SSDI
- 7 – A Student (full-time)
- 8 – A homemaker
- 9 – Retired
- 10 – Other – please specify: ______________________

**4. What is the zip code where you currently reside?** ______________

**5. We have in our records that you are living with Hepatitis C or HCV. Is this correct?**

- 1 – Yes
- 2 – No
- 88 – I don’t know.

**6. How many miles do you currently travel, one way, to get your HCV Care?** _______________Miles

**7. Which gender describes you? (Read all options)**

- 1 – Man
- 2 – Woman
- 3 – Transgender (if Veteran identifies as transgender, then ask them to specify either 3a or 3b.)
- 3a – Male to Female
- 3b – Female to Male
- 4 – Other, Please describe ________________

**8. What sex were you assigned at birth on your original birth certificate?**

- - - - 1 – Male
      - 2 – Female
      - 99 – Decline to answer

**9. Is your ethnic background Hispanic or Latino?**

- 1 – Hispanic or Latino; specify _____________
- 2 – Not Hispanic or Latino

**10. What is your highest level of education?**

- 1 – 8th grade or less
- 2 – Some high school
- 3 – High school graduate/GED
- 4 – Technical school
- 5 – Some college
- 6 – College graduate, Bachelor‟s degree
- 7 – Master‟s Level Degree – e.g. MA, MS, MPH, etc.
- 8 – Doctoral Degree - e.g. MD, PhD or similar

**11. Do you think of yourself as:**

- 1 – Lesbian, gay, or homosexual
- 2 – Straight or heterosexual
- 3 – Bisexual
- 4 – Something else please describe _______________
- 88 – Don't know

**12. What is your current living situation?**

- 1 – Living on your own
- 2 – Living with a spouse or domestic partner
- 3 – Living with friends
- 4 – Living with parents/family of origin
- 5 – Living with roommates or housemates
- 6 – Staying with people temporarily
- 7 – Other – please specify: ________

**13. What is your marital status?**

- 1 – Married
- 2 – Living with someone as though married
- 3 – Separated
- 4 – Divorced
- 5 – Widowed
- 6 – Single, never married
