# Supplementary material for: The health equity implementation framework: proposal and preliminary study of hepatitis C virus treatment
Source: Implement Sci. 2019 Mar 12;14:26. doi: 10.1186/s13012-019-0861-y (PMC6417278; doi:10.1186/s13012-019-0861-y)
Supplement: Supplementary file 4 — Interview Guide for HCV Treatment Implementation Assessment Informed by Health Equity Implementation Framework. This is the interview guide used in the preliminary study with patient participants that was aligned with the Health Equity Implementation Framework and showcased questions specifically about disparities. See the discussion section for limitations and advantages of using those questions about disparities. (DOCX 26 kb) [file 13012_2019_861_MOESM4_ESM.docx]

**Additional File 4.**

Interview Guide for HCV Treatment Implementation Assessment Informed by Health Equity Implementation Framework

1. **KNOWLEDGE OF HCV TREATMENT: *Have you heard about the new Hepatitis C treatment? What do you know about new treatment?***^[[1]](#footnote-1)^
   1. They come in the form of pills, not shots. You get them from your liver or GI doctor at VA. Sometimes they are called antivirals (examples below).
      1. Epclusa (sofosbuvir and velpatasvir) – pink, diamond shaped pill
      2. Zepatier (elbasvir and grazoprevir) – yellow, cream-colored oval pill “770” on it
      3. Harvoni – pink, orange-ish diamond shaped pill, “GSI” on it
      4. Sovaldi, (sofosbuvir) – beige, cream circle pill, “GSI” on it
      5. Olysio (simeprevir), **sometimes ribavirin.** I will call this the new Hep C treatment.

[Information about Hep C Treatment:

There are new treatments that can cure Hepatitis C, to the point where when your blood is tested for Hep C, the results will be clear—no Hep C will be found in your blood. This new Hep C treatment has the potential to cure hepatitis C after about 12 weeks. Over 90% of Veterans treated with the new Hep C treatment have achieved a cure. The treatments are only pills (not shots or injections) with few side effects. The VA currently offers this to Veterans with Hep C.

They have been invented in the last 5 years or so. Other new treatment names: direct-acting antivirals (DAA), or interferon-free therapy, another med = elbasvir. Old Hep C treatment = Triple therapy = two pills + interferon injections or just interferon injects.]

- 1. Have you heard of having a genetic blood test done to see which type of Hep C you have?

[For reference: Types 1, 2, 3 (most people in the U.S.); Types 4, 5, 6 also exist. Treatment differs depending on which genotype a person has.]

- 1. Have you ever learned what stage of liver disease you have, if any? (examples: Stage 1, 2, 3…)

*Thank you for telling me about that. Next, I’m going to ask you more in detail about your Hep C treatment.*

1. **THEIR TREATMENT EXPERIENCE: *When were first told you had Hep C?***^[[2]](#footnote-2)^1
   1. What treatment have you been offered for Hepatitis C?

Probes: Do you recall talking to anyone about Hep C?

Did you start taking the pills for new Hep C treatment?

Did you complete a course of Hep C treatment?

In VA or outside VA?

[For reference, new Hep C treatment requires getting follow up screenings to ensure you’re “clear” of Hep C.]

1. **If no to getting new Hep C treatments:**
   1. No treatment at all, or just not completed an entire course of it? If you’ve taken even a single pill of the new treatment, that counts as starting treatment.
   2. Have you ever learned what stage of liver disease you have, if any? (examples: Stage 1, 2, 3…)
2. ***BARRIERS/FACILITATORS: Thank you for sharing all that information about your experience. Next, we would like to share some lists of things that other Veterans have said makes it harder to get the new Hep C treatment, and see what you think about it. Are you ready?***

The first list is about things that stand in your way of getting the new Hep C treatment—things that make it harder to get new Hep C treatment or reasons why you have not completed a course of new Hep C treatment. Remember: these are all about Hep C treatment, not treatment for other conditions like blood pressure or diabetes. Please say yes if you have experienced any of these troubles and no if you haven’t.

| **LEVEL** | **LIST OF BARRIERS**  **(ask each one)** | **FACILITATORS** |
| --- | --- | --- |
| **Innovation**  **Clinical Encounter** | **This first list is about when you meet with doctors and the treatment itself.**  **The Treatment Itself**   1. Anything about taking pills 2. Cost of medicine 3. Cost of appointments   **Other Innovation Factors**   1. Hard to get an appt soon 2. Knowing the new treatment existed 3. Ability to try the treatment out first 4. The new treatment didn’t seem better than old treatments 5. Wait times to get an appointment 6. Waiting in the clinic the day of your appointment 7. Previous negative experience with Hep C treatment 8. Anything else about the Hep C treatment?   **Clinical Encounter**   1. Problems when you met with a doctor or nurse 2. Bias: Ever felt treated differently in getting treatment for Hep C 3. Not being understood by your doctor or nurse 4. Doctors/nurses seemed like they were “keeping their distance” from you because you had Hep C 5. Anything else about meetings with doctors or nurses? | **How did you overcome (barrier)? What did you do then? Did anyone or anything make (barrier) easier?**  **The Treatment Itself**  **Other Innovation Factors**  What has been the best place for you to get information about Hep C treatment?  **Clinical Encounter** |
| **Recipients** | **This second list involves things about you and your preferences.**  **Patient Factors**   1. Any biological issues – wrong genes/ genotype or other diseases that made it hard to get treatment 2. Not having any symptoms of Hep C 3. Your preferences for treatment 4. You felt like you couldn’t ask or insist on getting the treatment 5. Your motivation to get new Hep C treatment 6. Stress or depression in your life 7. Housing or your living situation 8. Don’t have enough money to get treatment 9. How your cultural group views Hep C 10. How your cultural group views hospitals, medicine, or doctors 11. Trusting your doctors and nurses 12. Stigma about having Hep C – feeling ashamed or guilty 13. Hard to understand all the parts of the new treatment to help you make a decision 14. Negative things you heard about the treatment from other people 15. Distance from hospital/clinic 16. Transportation 17. Anything else about you?   **This third list includes things about your doctors and nurses.**  **Provider Factors**   1. Too busy 2. Doctors did not believe the new treatment would work 3. Doctors said you should not get the treatment 4. Doctors were supposed to call about the treatment and they didn’t 5. Didn’t answer your questions 6. Didn’t seem to respect you or your visit 7. Anything else about your doctors or nurses? | **Patient Factors**  For you, have you ever had any good experiences with friends or family helping you through Hep C treatment? Or would you like that?  **Provider Factors** |
| **Context** | **This next list includes things about your local hospital where you get most of your health care.**  **Inner Context (local level)**   1. Doctors/nurses did not ask for your opinion or give you a choice 2. Bad experiences at that clinic with other medical conditions 3. No doctors to give you the treatment at your local hospital 4. Harder to get treatment at your local hospital 5. Anything else about your local clinic or hospital?   **This next list is about the VA, in general.**  **Inner Context (organizational level)**   1. Did you ever use or try to use Veterans Choice Program?    1. Too complicated    2. Were not able to 2. The VA doesn’t seem to try new things or use best medicine 3. The VA doesn’t seem like they wanted to help patients very much 4. The VA has not offered the treatment 5. The way the VA wants you get treatment doesn’t work for you (e.g., coming in regularly for check-ups) 6. Anything else about VA?   **This last list of things is about environment and society.**^[[3]](#footnote-3)^  **Outer Context**   1. Lack of caring about patients living with Hepatitis C 2. Lack of caring about patients who are not white 3. No media coverage on TV or radios or signs about new Hep C treatment 4. Anything else about society or environment, at large? | **Inner Context (local level)**  **Inner Context (organizational level)**  **Outer Context**  Do you feel like information you see about Hep C or treatment for Hep C applies to people like you? |

**Open Ended Health Disparity Barriers Question**

On the questionnaire you stated you were (insert Black or African American). How does that relate to getting the new Hep C treatment? Did you ever feel you were treated differently trying to get the new Hep C treatment?

**Open Ended Health Disparity Facilitators Question**

Did being (insert Black or African American) ever make it easier to get the best Hep C treatment? How?

1. **IDEAS ABOUT TREATMENT.** My final set of questions is about how you think the VA could improve new Hep C Treatment. Besides things you’ve already shared with me, specifically, what would you want to see the VA or the VA providers do to better treat Veterans living with Hep C?^[[4]](#footnote-4)^

- Is there anything else you’d like to add?^[[5]](#footnote-5)^
- Do you have any concerns or questions for me at this time?

1. Author note: The first two sections were created to understand patients’ current experience with HCV treatment and also to build rapport. They were not directly aligned with the framework. [↑](#footnote-ref-1)
2. 1 Author note: The first two sections were created to understand patients’ current experience with HCV treatment and also to build rapport. They were not directly aligned with the framework. [↑](#footnote-ref-2)
3. Although i-PARIHS and the Health Care Disparities Framework limit contextual determinants to be within the healthcare system, we expanded the outer context to consider societal influence given the condition (HCV) and vulnerable population (Black VA patients) of inquiry. [↑](#footnote-ref-3)
4. This question was added at the request of our operational partner to generate a list of recommendations for VA about HCV treatment. Those recommendations are outside the scope of this manuscript and not presented here. [↑](#footnote-ref-4)
5. This is an example of one question that garnered responses about how HCV treatment might be specific to Black or African American patients. Participants would often discuss those culturally relevant details more openly in response to this question, possibly because they had been primed to consider race and culture in the health disparity questions that preceded this question. [↑](#footnote-ref-5)
